# Periscope

**Published:** 1887-09

**Authors:** 


### PERISCOPE.

### PHYSIOLOGY.

**The Stomach.**—Some important points in the anatomy and physiology of this organ, and their application in practice, are discussed by A. W. P. Leuf in the *Philadelphia Medical News*, April 16th, 1887. The following is a summary of his conclusions:—(1) This viscus is more nearly vertical than horizontal in position; (2) A healthy stomach when empty is tubular in form; (3) Simple, unirritating liquids pass directly through the tube; (4) also, if the stomach contains food such liquids pass through at once along the lesser curvature; (5) Digestion in the morning is hindered by the presence of mucus in the stomach; (6) Water drunk before meals dilutes and washes out this mucus, stimulates peristalsis, and causes hyperæmia of the lining membrane, thus aiding both digestion and elimination; (7) Cold water is best for those who have good power of reaction, but warm or hot water for others; (8) the addition of a little salt prevents the formation of unabsorbable parapeptones; (9) finally—water, drunk before, during, or after meals, does good rather than harm.

**Biological Action of Copper.**—In *A Medicina Contemporanea* for April and May, 1887, Dr. Curci publishes his researches on the above. He finds that in frogs copper causes paralysis of the cerebral motor centres and of the organs of circulation. In dogs it causes first paralysis of the reflex centres of the cord, then of the cerebral sensory centres, and lastly of the cerebral and spinal motor centres. Reflex action and sensibility to pain are abolished before motility is affected. Copper at first stimulates the intracardiac excito-motor nervous apparatus and the vaso-motor system generally, and causes increased arterial pressure; it then paralyses them and causes lowering of arterial pressure. It lowers the temperature by dispersion of heat. This, and the circulatory paralysis, account for the adynamia and collapse in cases of poisoning, of a certain duration, by the salts of copper. Death is caused by respiratory paralysis, pulmonary œdema depending on vaso-motor paralysis, and cardiac paralysis. Copper is firstly and chiefly a nerve poison. Injected into the veins or hypodermically it does not cause vomiting.—*London Medical Record*, August, 1887.

ARTHUR B. PROWSE, M.D.

### MEDICINE.

**Endemic Goitre.**—A paper on the above, embodying observations made during a visit of some months' duration to the high tableland of central Asia, N.W. of Cashmere, by Surgeon G. M. Giles, M.B., F.R.C.S., of H.M.I. Service, while in charge of the C.K. Mission, was published at Calcutta on Dec. 14th, 1886. This affection is common throughout a large portion of the N.W. Himalayas and Hindu-Kush mountains, and the proportion is at least 5 per cent. of the population. The majority of cases commence about puberty; but no age is exempt from the incidence of the disease. Between 12 and 13 months is the average time of residence for individuals from other districts before a goitre is developed. The prevalence of the affection among these mountains is, in most cases, roughly inversely proportional to the plentifulness of trees; but in one district, almost bare of forest growth, it is even less common than in the well-wooded parts. Now, where trees are plentiful, the rude cots of the natives are built of it, and are, perforce, much better ventilated than the dark and close mud huts of districts where wood is scarce. In the district above-mentioned, which is nearly exempt, notwithstanding the want of wood, the houses are built of stone, and are larger, and, moreover, are well ventilated by the central openings (four or five feet square) in the roofs, which serve the purpose of chimneys. "In spite of all that has been written and sung of the 'bold mountaineer,' the struggle for existence in such regions is so severe, that he is generally much more poorly off than the dweller in the plains, and his house and immediate surroundings proportionately insanitary." The Kashmiri garrison at Gilgit in two years have attained to an average of goitre probably rather higher than that of the indigenous population; and this may, doubtless, be ascribed to the fact that the fort is much the most crowded and insanitary portion of the village. Insanitary homes cannot, however, be more than a predisposing cause of goitre, for the wretched inhabitants of London cellar-tenements live in houses at least as crowded and quite as insanitary as those of the poorest mountaineers of Dardistan. Being on the march, the author's appliances for microscopic research did not go beyond 250 diameters, and he necessarily had no conveniences for culture and inoculation-experiments; but from observations with the 'power' mentioned he is inclined to think that a species of low vegetable organism is the exciting cause of the disease. The general course of a goitre is to go on growing to a considerable size, and then to remain stationary; but a by no means insignificant number of cases, after reaching a certain size, undergo a spontaneous cure. The cysts appear to dry up, and the tumour to contract, leaving a number of nodules about the size of walnuts, and of cartilaginous hardness.

In three months the author treated more than 150 cases by the injection of liquor iodi, B.P. The method of procedure was as follows:—"The tumour is first carefully examined, so as to make out the number and position of the various cysts or lobules; the needle of the syringe is thrust into each of these in succession, and about twenty drops of the solution injected. If the lobes are large, soft, and not distinctly cystic, a small dose is injected into two or three portions of each." One skin-puncture only is necessary as a rule; and pain is exceptional, either during or subsequent to the operation. One sitting is often sufficient to cause a cure; but a weekly repetition of the injection will hasten matters. Under this treatment the goitre steadily shrinks, until nothing but a number of hard nodules are left, which, being very slightly vascular, might very well be excised subsequently. The author considers that the treatment involves no appreciable danger if a *watery* solution of iodine be used.

**Should the Pleural Cavity be washed out?**—This is discussed in a practical manner by Dr. Basil in the *Medical Chronicle*, Aug., 1887. He points out how very diverse is the teaching of standard medical and also surgical authors on this question. An epitome of some of the best recorded cases in which a fatal result has immediately supervened on the process then follows. In attempting to explain the causes of death or of dangerous seizures from pleural injections the author reviews—(1st) the broader question as to the causes of sudden death in pleural effusions, and (2nd) the conditions that tend to make the operation of injection dangerous or the reverse. The conclusions drawn are the following:—(1) When from the physical examination of the chest, the length of time the empyema has lasted, and the nature of the membrane binding down the lung, we feel sure that the chances of the lung expanding are very small, resection of an inch or two of rib, and thorough drainage by position, is the better operation, as being comparatively free from risk, doing away very much with the necessity of using injections, and also diminishing the risk of injections by the free exit it affords. (2) If the pus contain flakes, coagula, fibrinous pieces, or shaggy masses, we must generally wash out, unless resection is done at once. (3) Where from the general state of the patient recovery is hopeless, but emptying of the empyema is forced on one, injection may be used with care should septic absorption set in. (4) In certain cases it is not easy to get consent for resection, and we must, if the discharge is fœtid, run the risk of pleural injection. (5) When from carelessness the drainage-tube has slipped in, among other means, washing-out of the pleural cavity may have to be resorted to. (6) In the adult the operation is always a dangerous one, and in every case we must have a clear object in view for running the risks. (7) The fluids recommended are freely-diluted Condy's fluid, weak solutions of boracic acid or of creasote, and solution of corrosive sublimate 1 in 6000 or 8000. It is best to use an arrangement in which gravitation is the force employed to introduce the fluid, instead of using a hand syringe; and the temperature of the fluid should be as nearly as possible that of the body. (8) It is a question whether atropia should not be used as a prophylactic to ward off the danger of reflex cardiac inhibition, or as an antidote in case of unpleasant symptoms setting in.

**Adherent Pericardium.**—Two instructive cases are reported by Dr. F. W. Mott, in the *Practitioner*, February, 1887. In each the striking symptom was ascites, with some enlargement of the liver. In both, pulmonary symptoms were prominent, pointing to old and recent pleurisy, with adhesions and thickening. In neither was there any history of acute rheumatism, and in both the disease seems to have commenced as a pleuro-pericarditis. In neither did the dropsy appear until six months after recovery from the acute attack. Other symptoms were, absence of apex-beat and signs of cardiac failure apart from valvular disease. The ascites was only temporarily relieved by paracentesis, and the fluid re-accumulated with great rapidity. The urine was scanty, but not albuminous. The difficulties of diagnosis were in both cases unusual, and, in the absence of positive signs, could only be established by a process of exclusion. The *post-mortem* appearances in their main features were identical.

In an excellent monograph by Riegel, in Volkmann's *Sammlung Klinischer Vorträge*, 1879, he points out the importance of the weakness or absence of the apex-beat, both in diagnosis and prognosis ; also that a distinct systolic recession indicates abnormal movement of the heart and pericardial adhesion, but it shows that the heart is acting powerfully and that there is either compensatory hypertrophy, or at any rate that the muscle-substance has not undergone degeneration. The prognosis, therefore, depends much upon the presence of this sign ; for as the strength of the heart diminishes owing to degeneration, so this sign disappears.

**Friedrich's Disease.**—In *Le Progrès médical*, June 4th, 1887, Charcot has an able clinical lecture on the differential diagnosis between this disease and tabes on the one hand, and disseminated sclerosis on the other. He presented two patients, the younger of whom had suffered from Friedrich's disease for ten years, the elder from tabes for a similar period. In both there was marked motor inco-ordination of the upper extremities. In the lower limbs there was no loss of muscular power, and the patients could stand without support; but there was instability when the feet were approximated, or the eyes were shut, and both had the tabetic gait, and in both the knee-jerk was absent. The points in which they differed were as follows: The tabetic patient when seated was perfectly at rest, while the other was perpetually moving, the movements being involuntary and slow and resembling those seen in athetosis. The head oscillated from side to side or nodded, and from time to time there were grimaces. In the tabetic case there were also diplopia, strabismus, and sometimes drooping of the left eyelid. There was no atrophy of the optic nerves, but the Argyll-Robertson sign was well-marked. In the case of Friedrich's disease the pupils re-acted to light and to accommodation, they were equal, and there was neither diplopia nor strabismus; but there was some degree of nystagmus, a phenomenon very exceptional in tabes, but usual in disseminated sclerosis. The patient presented also another symptom belonging to the last-named disease; viz., slow articulation, and *scansion* of the words.

The case might possibly be regarded as one of tabes with disseminated sclerosis, or as a disseminated sclerosis with sclerosed areas in the posterior columns, giving rise to tabetic symptoms; but Charcot controverted both these views. Summing up the symptoms under their various systems—in Friedrich's disease there are not the lightning-pains, the hyperæsthesias or anæsthesias, nor the atrophy of the optic nerves, which are present in tabes. Neither are there the visceral neuroses nor the trophic lesions. It differs from disseminated sclerosis in having neither the vertigo, nor the epileptiform attacks, nor the white atrophy, nor the optic neuritis. The choreiform instability does not cease during repose, and it is rhythmic, not spasmodic or jerky; the motor difficulties are not influenced by sight; and the knee-jerk is absent, not exaggerated.—*Edinburgh Medical Journal*, August, 1887.

**Koprostatic Reflex Neuroses.**—The most frequent of these, says Kisch (*Berliner klin. Woch.*, No. 15, 1887), is cardiac neurosis, evidenced by palpitation, and in some cases by vertigo and irregularity of the pulse. Next comes hemicrania. Difficult as its pathogenesis is to explain, it is undoubtedly often due to chronic constipation. Sciatica is another frequent result, to which various lumbo-abdominal and ovarian neuralgias are allied. Prof. Gussenbauer (*Präger med. Woch.*, No. 31, 1886) has already mentioned neuralgia of the fifth nerve in this connection. Of twenty-eight cases, all but four were cured by rectifying the constipation.—*London Medical Record*, July, 1887.

ARTHUR B. PROWSE, M.D.

### SURGERY.

**Unusual Site of a Hydatid Cyst.**—A case of hydatid of the scrotum came under the care of Mr. Philip Muskett, in the Sydney Hospital. The patient, aged 25, had lived in a sheep-district, where the water was very bad, four years before the swelling appeared. This was first noticed eight years before he came under Mr. Muskett's care. During that time the swelling had been twice tapped, and on the second occasion injected with iodine. When admitted to the hospital there was a large intra-scrotal tumour, the size of an emu egg, on the right side. This was translucent, and presented the usual characteristics of a hydrocele. The cyst was tapped, and an ounce of pale yellowish fluid drawn off. Suppuration followed; and thirteen days later, six ounces of pus were drawn off. After this, pus drained away from the puncture for eleven days; then a hydatid sac partly forced its way out, and was partly extracted through this opening. The sac was greyish in colour, translucent and elastic; and in its sunken condition would about fill an egg-cup. The swelling gradually subsided, the sinus closed, and the patient left cured.—*The Medical and Surgical Reporter*, Feb. 26th, 1887.

**The Treatment of Hernia by Subcutaneous Injection.**—At a meeting of the New York Medical Society, Dr. De Garmo brought forward the subject of radical cure of hernia. During the last seven years he had treated over 100 cases by Heaton's method of injecting astringent and mildly irritating fluids, such as a solution of oak-bark, into the inguinal canal outside the sac. The object is to fortify the fibrous tissues surrounding the canal, that protrusion should not occur. Dr. De Garmo complained that the method had been put to unfair tests. Patients had been allowed to go about without support, after rising from the bed, seven to ten days after the operation. Another error was the anticipation of cure in old hernia by a single injection. The most favourable cases were those of oblique inguinal hernia of recent date, in which but few pathological changes had occurred.

He concluded from the results of his cases treated on this plan, that the operation is free from danger. That over 45 per cent. of all cases could be cured by it; and in selected cases, 50 to 75 per cent. That many extreme cases, uncontrollable by a truss, could be brought under control by the operation. That it was followed by improvement in almost every instance. That children not cured by mechanical means could in almost every instance be cured by this method.—*New York Medical Journal*, Feb. 19th, 1887.

**Rectal Injections in Cystitis and Enlarged Prostate.**—Dr. Vercoe calls attention to the value of rectal injections of hot water in cases of enlarged prostate and cystitis. He irrigates the prostate and base of the bladder with hot water, applied directly to the parts through a large double-eyed catheter passed per anum.—*The Medical Age*, Aug. 10th, 1887.

[This very simple treatment, often of great value, has not received the attention it deserves.]

W. J. PENNY.

### THERAPEUTICS.

**Nitro-glycerine in Mitral Stenosis.**—In the *Liverpool Medico-Chirurgical Journal*, July, 1887, is an interesting paper by Dr. Barr on the treatment of cases of mitral stenosis. He says that there is no single remedy of such value in these cases as nitro-glycerine, but is careful to explain that he does not mean that it is a universal panacea for all cases and complications, or that its use is always necessary or advisable. The object aimed at is "to equalise the disturbed balance of circulation and to lessen the high pulmonic tension," thus enabling the right ventricle to perform its work more thoroughly. This is effected by the dilatation of the systemic vessels by the drug, and by limiting the amount of fluid ingested, by moderate catharsis, by passive exercise of the muscles, and by active exercise when the patient is fit for it.

[I have had under observation for some months in the out-patient department of the Bristol Royal Infirmary a young woman, the subject of considerable stenosis of the mitral orifice. The persistent impediment to the circulation is clearly evidenced by the state of the extremities, especially of the fingers, which are always more or less cold, livid, swollen and painful. In this case nitro-glycerine always produces considerable amelioration of these and other symptoms. The dose given has never exceeded $\frac{1}{100}$ of a minim thrice daily.]

ARTHUR B. PROWSE, M.D.

**Benzoin in the Treatment of Ulcers.**—In the *Russkaia Meditzina*, No. 35, 1886, Dr. Voskresensky speaks highly of benzoin as a specific remedy for treating ulcers of all kinds, especially those which occur in emaciated, cachectic, and old persons.

The author employs it in the shape of a salve, prepared *ex tempore* of two drachms of resina benzoes, half an ounce of yellow wax, and half an ounce of lard.

The ointment is spread in a moderately thick layer on a piece of linen, and placed on the ulcer. The dressing, as a rule, should be changed twice a day, but in bad cases three times daily.—*Medical and Surgical Reporter*, Feb. 19th, 1887.

W. J. PENNY.

### DERMATOLOGY.

**Alopecia Areata.**—The question whether this affection is a parasitic or nervous one is of practical importance. Dr. Wyss, of Geneva, reports (*Der Fortschritt*, Jan. 5th, 1887) the case of a child in which the patches were strictly localised to the left half of the scalp. Dr. Joseph (*Monat. f. prakt. Dermat.*, Nov., 1886) recently made experiments with cats on the etiology of the disease, in Du Bois-Reymond's Physiological Institution at Berlin. He observed, after the division of the spinal ganglion of the second cervical nerve, in every case a depilation analogous to that of the disease, without any previous inflammatory symptoms on the circumscribed patches of skin. He, therefore, considers the disease a lesion of the trophic cutaneous nerves. Buchin states (*Monat. f. prakt. Dermat.*, No. 10, 1887) that there are striking instances on record of alopecia areata having been produced epidemically. He quotes examples from Gillette and one from Nogent, in which the epidemic attacked 400 children, and also a number of recruits in the 122nd Line Regiment (French).—*London Medical Record*, June and Aug., 1887.

[In connection with these quotations from French authorities the thought suggests itself that they use the term alopecia areata in a much wider sense than we are accustomed to.]

ARTHUR B. PROWSE, M.D.

### OPHTHALMOLOGY.

**Sympathetic Ophthalmitis.**—A good example of the value of co-operative work is afforded by the "Report" on this terrible affection, based upon the notes of 200 cases. The conclusions (in addition to facts included in text-books) are as follows:—(1) Sympathetic ophthalmitis never occurs without *perforation* of the exciting eye, either by wound, or ulceration, &c. (2) It generally occurs within one year of the injury. (3) It may occur at a greater interval, provided recurrent inflammation affects the injured eye. The interval between the last recurrence and the onset of the affection in the sympathising eye would be less than twelve months. The longest interval after the primary injury was twenty years. (4) The shortest interval reported was nine days. (5) Excision of the exciting eye, after sympathetic mischief has set in, does not apparently affect the progress of the disease one way or the other. (6) The effect of mercury in these cases is *nil.* (7) Iridectomy on the exciting eye, after commencement of the sympathetic mischief, in a few cases, has appeared to produce a favourable result. On the sympathising eye the results were less unfavourable than have generally been supposed. (8) In cases where the disease has appeared after excision of the exciting eye, the disease was due to the condition of the eyeball, and not to the operation.—*Liverpool Medico-Chirurgical Journal*, Jan., 1887.

**Retinal Hæmorrhage in Anæmia.**—An important observation in regard to this has been made by Dr. Stephen Mackenzie. It has for some time been recognised that in "pernicious anæmia" these hæmorrhages occur, and they have been to some extent relied upon as diagnostic of this intractable disease. But by observation of cases of anæmia secondary to loss of blood, Dr. Mackenzie concludes that the hæmorrhages are indicative rather of the degree than the nature of the anæmia. The blood was examined in reference to the number of red corpuscles, and the amount of hæmoglobin. When the corpuscular richness falls below 50 per cent. of the normal, a condition of retinal danger is established. As a clinical test he states that as long as any pink colour can be seen through the finger-nails, the corpuscular richness is above 50 per cent. The quantity of hæmoglobin exercises a qualifying influence on the main factor, according as it is greater or less than the corpuscular deficiency.—*Liverpool Medico-Chirurgical Journal*, Jan., 1887.

Arthur B. Prowse, M.D.

### LARYNGOLOGY.

**Statistics of Croup and Diphtheria.**—From the reports of thirteen children's hospitals in Germany, Austria, and Russia, it appears that in 1884 there were admitted 1234 cases of croup and diphtheria, of which 556 died, a mortality of 45·05 per cent.

During the same period tracheotomy was performed in 438 cases, of which 164 recovered, a percentage of 37.44.

The plan of treatment in regard to tracheotomy seems to have varied widely at the different institutions. Thus, at St. Anne's Hospital, Vienna, where there were 214 cases, and 78 deaths, tracheotomy was performed in 144 cases, of which 74 recovered (51.4 per cent.): while at the Elizabeth Hospital, St. Petersburg, there were 89 cases and 49 deaths; but tracheotomy was performed only once, and then unsuccessfully.—*Jahrbuch f. Kinderheilk.*, B. xxiv., H. 4, and *Edinburgh Medical Journal*, Aug., 1887.

Arthur B. Prowse, M.D.

### PATHOLOGY.

**Sensory Aphasia.**—At the Liverpool Medical Institute, Dr. Wiglesworth exhibited a brain taken from a female patient, aged 65, who had been the subject of the amnesic or sensory form of aphasia. She had been able to say numerous words and sentences, but could not apply them properly; was quite unable to name any common object, and, as a rule, even to recognise the name when told it, but could sometimes indicate its use. She could not read, nor could she write, either from a copy or from dictation. She was to a certain extent conscious of her defect, but clearly often did not understand what was said to her.

The left hemisphere presented a deep depression, occupying the situation of the inferior parietal lobule, and encroaching upon the temporo-sphenoidal lobe: this was due to the destruction and absorption of convolutions in this area, viz., the posterior three-fourths, or rather more, of the supra-marginal; the anterior three-fourths of the angular; and the posterior half of the first temporo-sphenoidal. The destruction extended from the surface into the white matter almost down to the lateral ventricle. It was apparently due to necrotic softening, owing to obstruction of the vessel supplying the area in question. All the cerebral arteries were considerably degenerated.—*Liverpool Medico-Chirurgical Journal*, Jan., 1887.

**Subcutaneous Ether Injections.**—MM. Pitres and Vaillard have published (*Le Progrès médical*, May 21st 1887) some notes on the effects which the subcutaneous injection of ether has produced on the nerves in its neighbourhood. They find that when the injection has been so directed as to approach the main nerve-trunks, it has set up after a few hours a neuritis in which the myelin first blends with the cylinder-axis, and then the whole nerve-structure becomes indistinct in a state of acute inflammation. The subsequent degeneration spreads downwards, never upwards, and re-generation begins in a special fashion. In fact, ether under these conditions produces an immediate necrosis of nerve-fibres; and it is just possible it may be of use in those cases where nerve-stretching is at present employed.—*Practitioner*, July, 1887.

**A Case of Hermaphroditism.**—Dr. M. F. Podestá, in the February number of *Los Anales del Círculo Médico Argentino*, describes the following case:—

One morning there came to the Italian Hospital a woman named Mary N., æt. 24. Italian by birth, of strong constitution, florid, and of medium stature; she at once attracted attention by her manly appearance, not at all in keeping with her female apparel. Her face lacked those smooth and correct lines that tend to produce beauty in woman, and which also accentuate the sexual expression; instead, the lines were hard and manly, her complexion bronzed, her upper lip covered with a black moustache such as a young man of twenty years might have, her hair was short, hard and unyielding to the female *toilette*, her forehead low, the mouth large with thick lips, eyes black and expressive, with long lashes and heavy eyebrows.

Altogether her features and general appearance had much more of the male than the female type. Her voice was rough, strong, and altogether wanting in the sweetness that characterises the female voice.

On being asked whether she was married, she bent her head, became confused, and then answered in the affirmative, adding that she had been married five years but had had no family.

She complained of some gastric trouble and painful micturition.

On examination of the genital organs a well-developed penis was discovered, having at its base two folds of thick skin well covered with hair and closely resembling the labia majora. The penis was of good shape, the glans being covered by a freely movable prepuce, the meatus urinarius was situated on the inferior face of the glans penis on a level with its mouth (or base), a case of true hypospadias.

The two thick folds of skin mentioned above were situated one each side of the perineum, and although they closely resembled the labia majora, they were evidently a divided scrotum without testicles. On separating these lips a smooth surface was found somewhat red towards the front and free from hairs, but not a trace of a vaginal opening. Pressure on this part with the finger gave a firm resistance, and was similar in character and seemed to form an integral part of the perineal plane.

By abdominal palpation and rectal examination, no signs of ovaries or uterus could be discovered.

Pressure on the urethra caused some purulent matter similar to blenorrhagic pus to escape.

The patient appeared to be one of those unfortunate beings on whom Nature seemed to have stopped undecided when on the point of setting the stamp that characterises the sex, thereby producing a mixture of man and woman.

The general appearance of the patient is as follows:—

Neck long, muscular, erect, manly.

Thorax manly, not a trace of mammary gland, very small nipples.

Chest-walls somewhat flat and hollow, slightly covered with hairs.

Respiration abdominal.

The pelvis has neither the diameter nor the anatomical arrangements that are observed in the female pelvis.

On placing the patient in the erect position, with the thighs together, the penis protrudes to the extent of three centimetres from beneath a well-covered pubes.

The attitudes and movements of the patient are manly.

The arms are strong, muscular, and covered with hairs.

There has never been a menstrual flow. Once only there was a slight loss of blood from the urethra.

J. Bain Sincock.

